# Subconscious pencil strokes in the HTP drawing test: a case study of an adolescent with emotional disorders

**DOI:** 10.1192/j.eurpsy.2026.11156

**Published:** 2026-07-31

**Authors:** C. Garcia Cerdan, M. Ligero Argudo, I. M. Peso Navarro, C. Munaiz Cossio, I. Rodríguez Blanco, A. I. Mitadiel Velasco, M. Benavides Madariaga, I. Sánchez Díez

**Affiliations:** 1 Psychiatry; 2 Psychology, University Clinical Hospital, Salamanca, Spain

## Abstract

**Introduction:**

The House–Tree–Person (HTP) test, developed by J. Buck in 1948, is a projective drawing technique widely used in child and adolescent psychology. Although projective tests have been criticized for subjectivity, they may offer valuable insights into emotional conflicts and self-concept in patients who struggle with verbal expression. This case study explores the interpretive potential of subconscious pencil strokes in the HTP test applied to an adolescent with emotional disorders.

**Objectives:**

To analyze subconscious drawing features in the HTP test and examine their clinical relevance by comparing the results with established interpretive frameworks and the patient’s psychiatric history.

**Methods:**

A 15-year-old second-generation immigrant female, admitted repeatedly to the Child Psychiatry Ward due to self-harm and suicide risk, underwent the HTP test. Her psychiatric diagnoses included adolescent-onset emotional disorders and mixed adjustment disorder. Drawings were assessed by two independent clinicians, focusing on stroke characteristics, placement, and symbolic content. A brief literature review was conducted to contrast findings with prior evidence.

**Results:**

**House drawing (Figure 1):** Placed in the center, without smoke, curtains, or flowers, and with two doors, reflecting emotional emptiness and ambivalence about belonging.

**Tree drawing (Figure 2):** Cut off on the left margin, suggesting impulsivity and preoccupation with past experiences. The wide trunk base indicates dependency, while the absence of fruit may reflect lack of fulfillment.

**Person drawing (Figure 3):** A faceless, hooded figure drawn from behind, without arms, symbolizing social withdrawal, isolation, and feelings of inadequacy.

**Stroke quality:** Across all drawings, lines were executed with a single, clean stroke, indicating decisiveness but emotional restraint.

**Size:** All three drawings were small, consistent with low self-esteem.

**Stroke quality:** Executed with a single, clean line, indicating decisiveness but emotional restraint.

**Symbolic content:** The house lacked signals of habitation (no smoke, curtains, or flowers), showing possible emotional emptiness. The tree displayed a strong trunk base (dependency) but no fruit (absence of fulfillment). The person was faceless, hooded, and without arms, consistent with social withdrawal, isolation, and feelings of inadequacy.

**Image 1:**
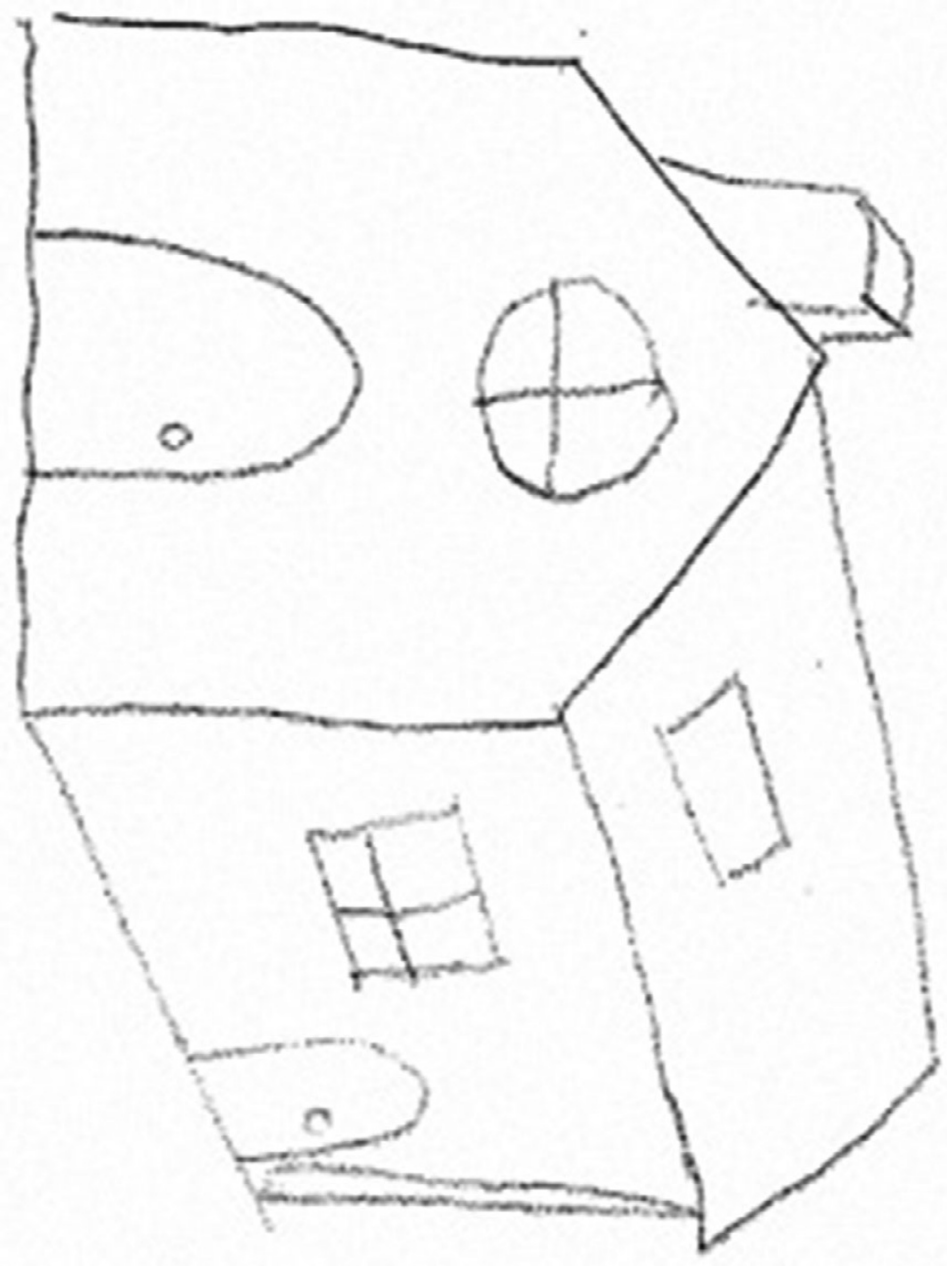

**Image 2:**
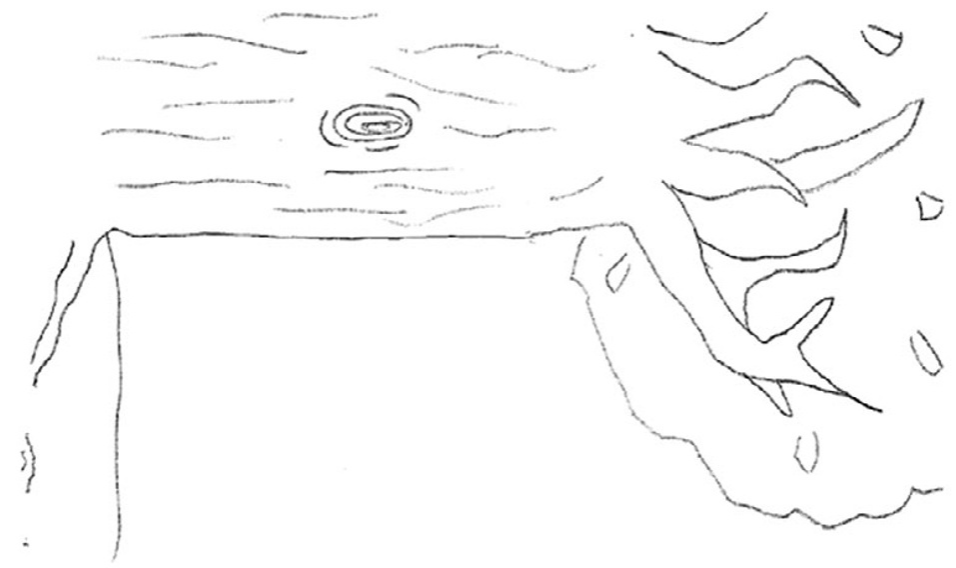

**Image 3:**
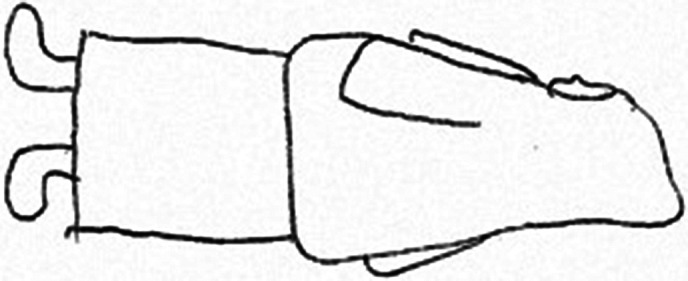

**Conclusions:**

The HTP drawings projected key aspects of the patient’s internal world, including limited sense of belonging, impulsive tendencies, and pronounced isolation. Although projective techniques should not be used in isolation, the HTP test may complement standardized measures by highlighting unconscious emotional conflicts. Future research should examine whether specific stroke patterns can be systematically correlated with psychopathological features in larger clinical samples.

**Disclosure of Interest:**

None Declared

